# Supplementary material for: Functional Deficits in Gut Microbiome of Young and Middle-Aged Adults with Prediabetes Apparent in Metabolizing Bioactive (Poly)phenols
Source: Nutrients. 2020 Nov 23;12(11):3595. doi: 10.3390/nu12113595 (PMC7700645; doi:10.3390/nu12113595)
Supplement: Supplementary file 1 [file nutrients-12-03595-s001.pdf]

Supplementary Material

Table S1. Intra- and inter- assay variations of metabolic status indices. [1]

	Glucose	Insulin	TC	HDL-C	TG
Intra-assay variation %	1.4	1.2	2.1	1.5	1.8
Inter-assay variation %	3.1	5.5	3.0	2.7	3.6

[1] TC, total cholesterol; HDL-C, high-density lipoprotein cholesterol; TG, triglycerides

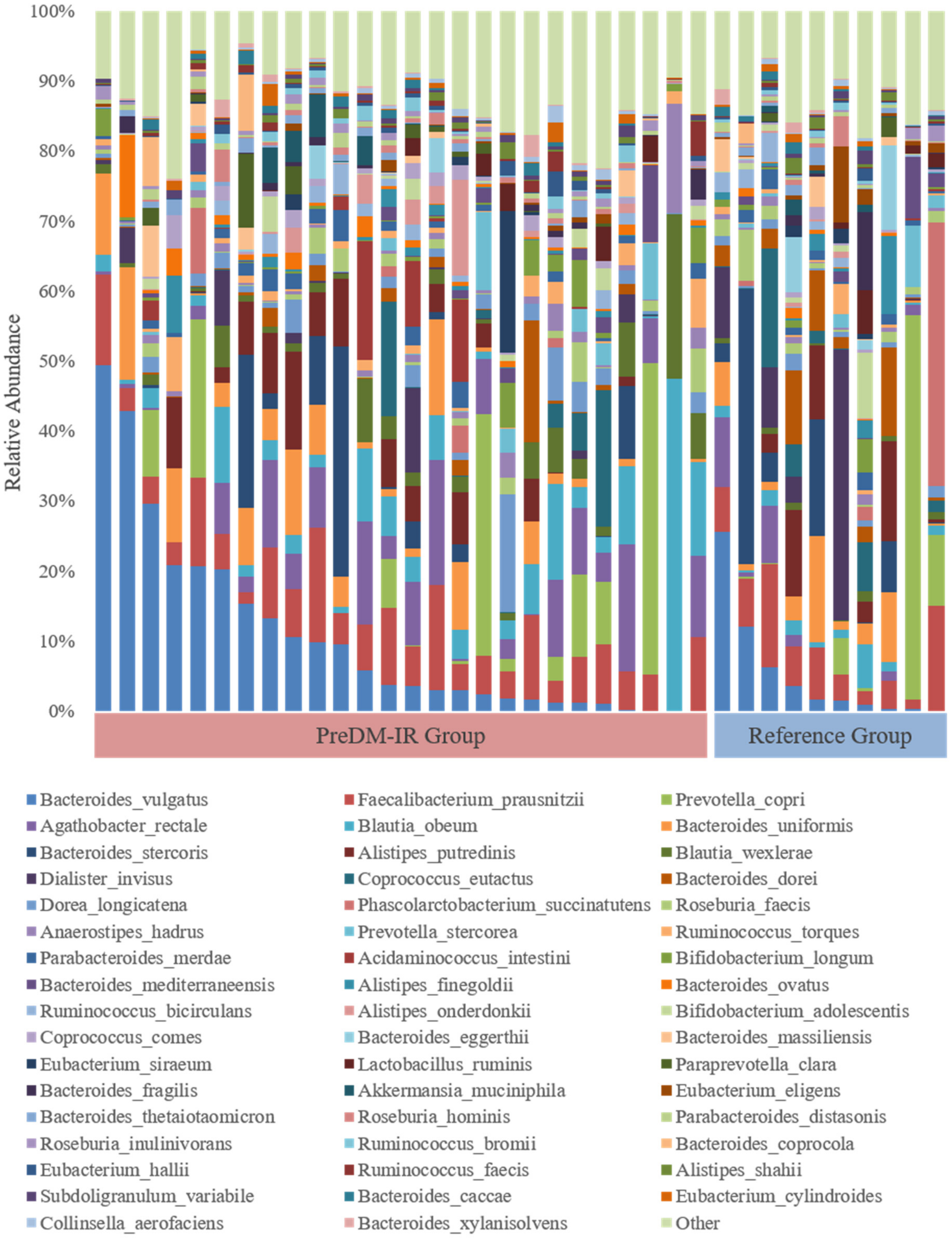

Figure S1. Gut microbiome composition at species level (top 50 species).

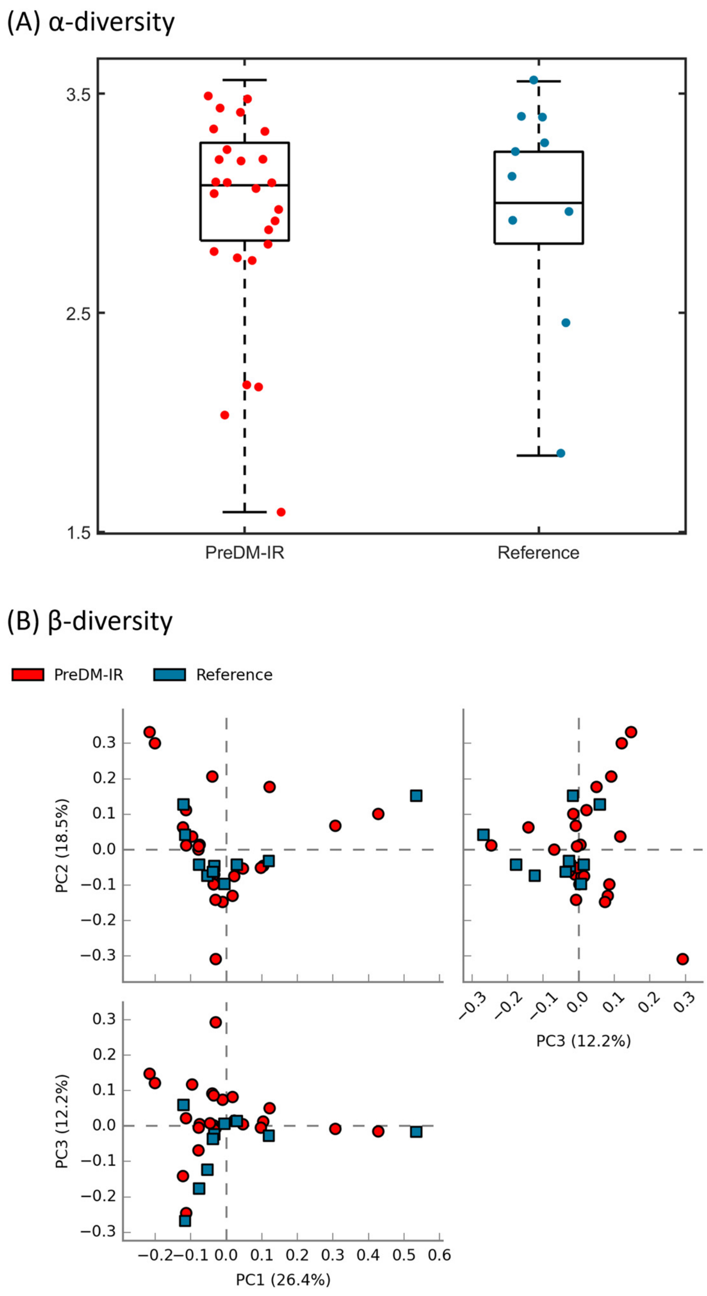

Figure S2. Within- and between- sample diversities: (A) α-diversity defined by the Shannon Index, indicating the number of species (richness) and relative abundance distribution (evenness) within a sample; (B) β-diversity defined by Principal Component Analysis (PCA), comparing species-level taxonomic profiles between PreDM-IR and Reference groups.

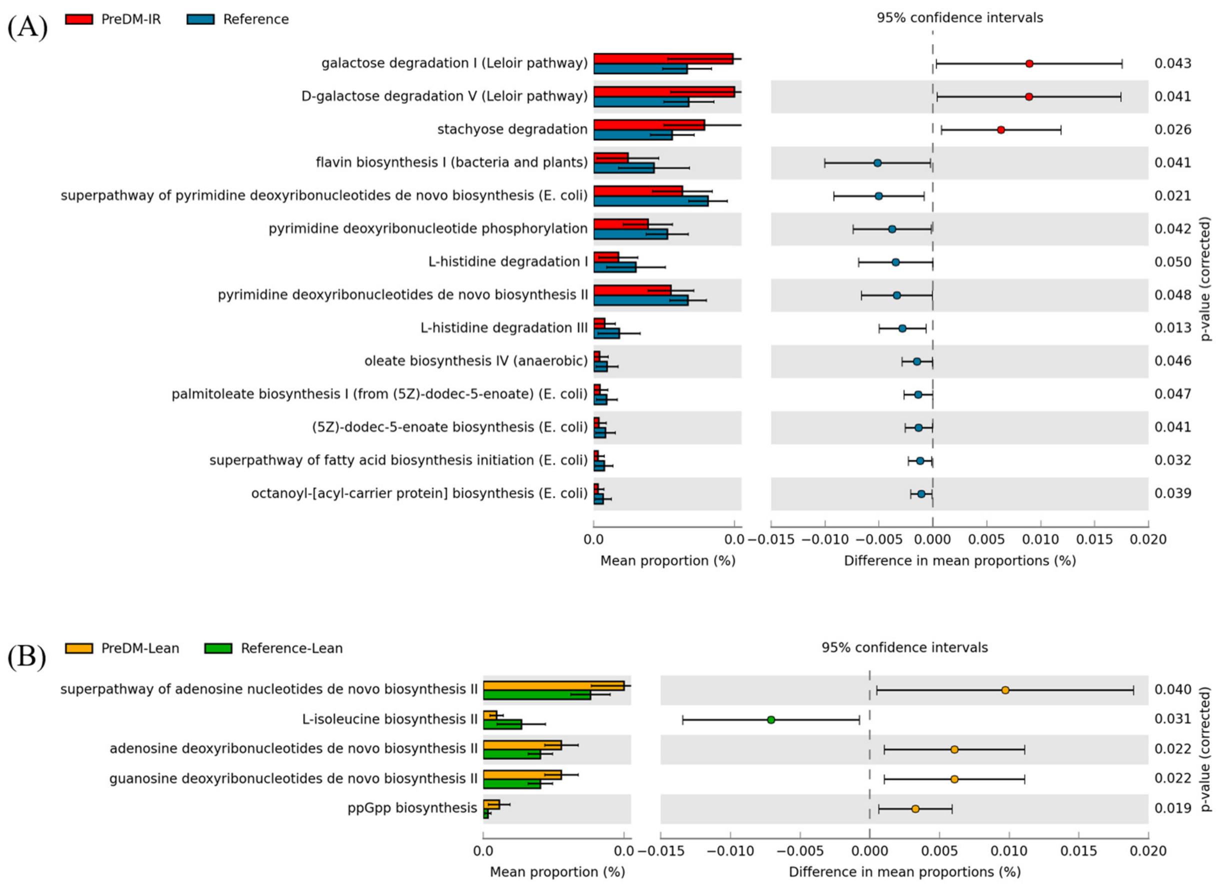

Figure S3. Predicted functional pathways: (A) PreDM-IR group vs. Reference group; (B) PreDM-Lean group vs. Reference-Lean group

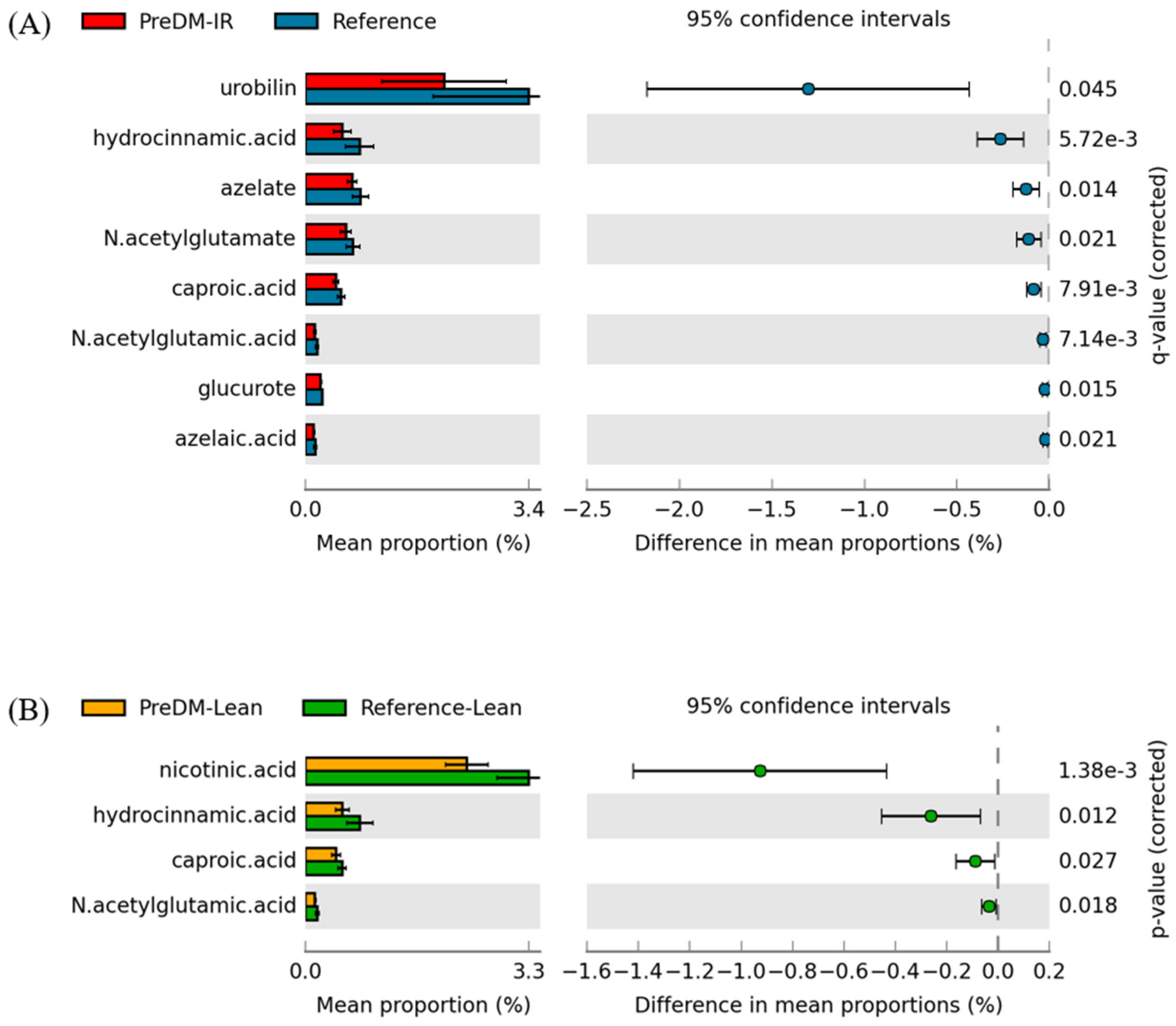

Figure S4. Predicted metabolomics: (A) PreDM-IR group vs. Reference group; (B) PreDM-Lean group vs. Reference-Lean group.
